# Gastro-intestinal tumours in rats and mice following various routes of administration of N-methyl-N-nitroso-N'-nitroguanidine and N-ethyl-N-nitroso-N'-nitroguanidine.

**DOI:** 10.1038/bjc.1969.94

**Published:** 1969-12

**Authors:** R. Schoental, J. P. Bensted

## Abstract

**Images:**


### GASTRO-INTESTINAL TUMOURS IN RATS AND MICE FOLLOWING VARIOUS ROUTES OF ADMINISTRATION OF *N*-METHYL-*N*-NITROSO-*N′*-NITROGUANIDINE AND *N*-ETHYL-*N*-NITROSO-*N′*-NITROGUANIDINE

R. SCHOENTAL AND J. P. M. BENSTED

*From the Toxicology Research Unit, Medical Research Council Laboratories, Carshalton, Surrey, and the Department of Biophysics, Institute of Cancer Research, Belmont, Surrey*

Received for publication June 5, 1969

MANY alkylnitroso compounds are effective carcinogens (Magee and Barnes, 1967) among which *N*-alkyl-*N*-nitrosourethanes are particularly interesting as they are able to induce tumours at the site of application without the need of enzymic activation.

*N*-methyl-*N*-nitroso-*N′*-nitroguanidine (MNG) (I), and its homologue *N*-ethyl-*N*-nitroso-*N′*-nitroguanidine (ENG) (II) resemble the respective alkylnitrosourethanes in some of their chemical and biological actions. Both these types of alkylnitroso compounds are decomposed by alkali to form the respective diazoalkanes. In the presence of free sulphydryls they undergo decomposition at pH 6·0–7·0, releasing nitrogen as gas and an alkylating moiety without the need of enzymes. It is not certain whether diazoalkane is an intermediary in this reaction with SH groups (Schoental, 1961, 1966, 1968 and unpublished results).

MNG has been reported to have an inhibitory effect on leukaemia (Greene and Greenberg, 1960; Goldin, Vendetti and Kline, 1959) and mutagenic action on various micro-organisms (Mandell and Greenberg, 1960; Eisenstark, Eisenstark and Sickle, 1965; Gilham, 1965; Lingens and Oltmanns, 1966; Siu, 1968) and viruses (Singer and Fraenkel-Conrat, 1967). It causes chromosomal aberrations in plants (Gichner, Michaelis and Rieger, 1963; Kaul, 1969; Müller and Gichner, 1964) and permanent bleaching of chloroplasts in *Euglena gracilis* (McCalla, 1965).

In rats, MNG induced tumours of the squamous part of the stomach when a few doses were given intragastrically (Schoental, 1966; Craddock, 1968*a*) and of the glandular part of the stomach when given continuously as dilute solutions in drinking water (Sugimura and Fugimura, 1967). On repeated subcutaneous injections, it induced local sarcomata (Sugimura, Nagao and Okada, 1966; Druckrey *et al.*, 1966).

The ethyl-homologue ENG is less active on the basis of both inhibition of ascites tumours (Greene and Greenberg, 1960) and of mutagenic action on *Arabidopsis thaliana* (Gichner and Veleminsky, 1967) but has not been so extensively studied. The carcinogenic effects do not appear to have been reported.

The respective alkylnitrosourethanes have been shown to induce mainly tumours of the stomach when given intragastrically (Schoental, 1963) but predominantly intestinal tumours after intraperitoneal injections (Schoental an Bensted, 1968). We now report similar dependence of the localisation of tumours when MNG or ENG were given by the intragastric or the intraperitoneal route to rats. MNG induced gastro-intestinal tumours also in mice.

### MATERIALS AND METHODS

Two strains of mice were used: C3H and CFW white. They were males, 1–2 months old at the beginning of the experiments, and were housed in metal cages, 6 per cage.

White rats, derived from the Porton Strain, bred randomly in the M.R.C. Laboratories, Carshalton, were used. Weanling males and females were separated according to sex and kept in metal cages about 5 rats per cage. They were weighed at the beginning of the experiment and at monthly intervals or more often. Both mice and rats were given the normal diet MRC 41B and water *ad libitum*. All the animals that died or that were killed by coal gas when they appeared ill, were autopsied, the livers, lungs, stomachs, kidneys and any other organs which seemed abnormal were fixed in Helly or neutral 10% formol saline solution; sections cut at 5–6 $\mu$ were stained routinely with haematoxylin and eosin for microscopic examination. Other stains were used when required.

The compounds MNG (I) and ENG (II) were commercial preparations supplied by the Aldrich Chemical Co., Milwaukee, Wisc. Both MNG and ENG were crystalline preparations, only sparingly soluble in ethanol or water and were used as suspensions in aqueous ethanol; the doses could therefore be estimated only approximately.

*N*-methyl-*N*-nitroso-*N'*-nitroguanidine
I

*N*-ethyl-*N*-nitroso-*N'*-nitroguanidine
II

### EXPERIMENTAL

The following experiments were performed:

A. *N-Methyl-N-nitroso-N'-nitroguanidine* (*MNG*)

1. Eight female and 6 male rats (50–90 g. body wt) were given a suspension of MNG in 30% aqueous ethanol by gastric intubation, 50–100 mg./kg. body weight.

In the course of the first year they were given additionally 1–4 doses not exceeding 50 mg./kg. body weight in a similar way.

2. Twelve male rats (40–55 g. body weight) were given MNG, by intraperitoneal injection, about 60 mg./kg. body weight in 0·1 ml. followed by additional 2–3 smaller doses 10–25 mg./kg. body weight within the first 11 months. Thereafter they were left without further treatment.

3. Twelve male mice, CFW were given 0·05–0·1 ml. of a suspension of MNG by intraperitoneal injections corresponding to approximately 1–2 mg./mouse. Two more similar doses were given 8 and 10 months after the first dose, respectively.

4. Twelve male C3H mice received similarly MNG, by intraperitoneal injection. Two doses of 2 mg./mouse each (100 mg./kg. and 60 mg./kg. body weight respectively) were given at 6 monthly interval.

5. Six male C3H mice were given a single dose of MNG 2·5 mg./mouse (125 mg./kg. body weight) by gastric intubation.

6. Six male C3H mice were given a single dose of MNG, 1·2 mg./mouse by subcutaneous injection.

B. N-*Ethyl*-N-*nitroso*-N′-*nitroguanidine* (*ENG*)

1. Four female and 6 male white rats (45–75 g. body weight) were given a suspension of ENG in 30% aqueous ethanol by gastric intubation 20–70 mg./kg body weight, followed by three additional doses given within 10 months.

2. Two females were given a single dose of ENG, 100 mg./kg. by gastric intubation.

3. Twelve male rats (40–55 g. body weight) were given by intraperitoneal injection suspensions of ENG, about 30 mg./kg. body weight followed by three additional doses not exceeding 20 mg./kg. body weight within 10 subsequent months.

4. Twelve white male CFW mice, about 1 month old, were given by intraperitoneal injection 1 mg. of ENU, followed by two more doses within 10 months.

5. Twelve male C3H mice were given two doses ENU by intraperitoneal injection of approximately 2 mg./mouse.

### RESULTS

A. N-*Methyl*-N-*nitroso*-N′-*nitroguanidine* (*MNG*)

*In Table I are summarised the dosage schedules and the times of survival of rats which had significant lesions.*

1. Of the 8 female rats all but one survived well over a year (1–2 years). Three rats had squamous cell carcinomata of the stomach with metastatic spread. One of these 3 rats showed, in addition, a highly anaplastic adenocarcinoma of the glandular portion of the stomach (Fig. 1, 2). Another of these rats had also a thymoma in the mediastinum (Fig. 3) and a further one had hyperplastic papillomatous changes in the squamous portion of the stomach.

An unusual multi-lobulated, polycystic appearance of the liver was observed in one rat (Fig. 4) associated with a large intraperitoneal (low grade) fibrosarcoma.

2. Among the 6 male rats, only 3 survived longer than 1 year (12–14 months) and all the three had squamous papillomata of the stomach (Fig. 5). Another had anaplastic peritoneal tumour (? primary).

3. Among the 12 male rats given intraperitoneal injections of MNG. the distribution of gastro-intestinal tumours was rather more widespread. Two rats had gastric tumours, 1 of which was an histologically proven squamous cell carcinoma. The jejunum of a third rat showed an adenocarcinoma whilst in 2 others the caecum was the site of a carcino-sarcoma and a round-cell sarcoma. A subcutaneous, very anaplastic sarcoma, was observed in another rat, possibly related to the needle track.

*In Table II are summarised the dosage schedules, and the times of survival of mice that had significant lesions.*

1. Among the 12 CFW mice, given MNG by intraperitoneal injections one had a vascular caecal tumour and another an adrenal cortical tumour. No gastric tumours were observed.

TABLE I.—*Significant Lesions Found in Rats Given* N-*methyl*-N-*nitroso*-N′-*nitroguanidine*

| Sex (total treated 26) | Dose (mg./rat) at times of treatment: 0 | 11 days | 28 days | 6 mth | 10 mth | Total dose (mg./rat) | Route | Survival (months) | Significant pathology |
|---|---|---|---|---|---|---|---|---|---|
| ♀ | 5 | 5 | 5 | 10 | 16 | 41 | i.g. | 11·5 K | Somach—warty papillomata |
| ♀ | 2·5 | 2·5 | — | — | 16 | 21 | i.g. | 15 K | Stomach—squamous cell carcinoma. |
| ♀ | 5 | 5 | 5 | — | — | 15 | i.g. | 20 K | Liver—multi-loculated, polycystic changes. |
| ♀ | 4 | 4 | 5 | 10 | 16 | 39 | i.g. | 24 K | Stomach—squamous carcinoma and adeno-carcinoma. |
| | 0 | | | 5 mth | 9 mth | | | | |
| ♀ | 5 | | | 10 | 16 | 31 | i.g. | 24 K | Stomach—squamous cell carcinoma, thymoma. |
| ♂ | 5 | | | — | 16 | 21 | i.g. | 10 D | Anaplastic peritoneal tumour. ?Primary. |
| ♂ | 5 | | | 10 | 16 | 31 | i.g. | 12 K | Stomach—warty papillomata. |
| ♂ | 5 | | | 10 | 16 | 31 | i.g. | 14 K | Stomach—warty papillomata. |
| ♂ | 5 | | | 10 | 16 | 31 | i.g. | 14 K | Stomach—warty papillomata. |
| | 0 | 6 mth | 9 mth | 11 mth | | | | | |
| ♂ | 3 | 3 | 5 | 12 | — | 23 | i.p. | 12 K | Caecum—round cell sarcoma. |
| ♂ | 3 | 3 | 5 | 12 | — | 23 | i.p. | 14 K | Jejunum—adenocarcinoma. |
| ♂ | 3 | 3 | 5 | 12 | — | 23 | i.p. | 15 K | Caecum—carcinosarcoma. |
| ♂ | 3 | 3 | 5 | 12 | — | 23 | i.p. | 15 K | Peritoneum—chronic fungal infection (?sporotrichosis) |
| ♂ | 3 | 2 | 5 | — | — | 10 | i.p. | 16 K | Subcutaneous sarcoma at injection site. |
| ♂ | 3 | 2 | 5 | 12 | — | 22 | i.p. | 18 K | Stomach—squamous cell carcinoma. |
| ♂ | 3 | 3 | 5 | 12 | — | 23 | i.p. | 19 D | Stomach—macroscopic tumour. |

TABLE II.—*Main Lesions Found in Mice Treated With* N-*methyl*-N-*nitroso*-N′-*nitroguanidine*

| Strain | Dose (mg./mouse) at times of treatment: 0 | 8 mth | 10 mth | Total dose (mg./mouse) | Route | Survival (months) | Significant/Pathology |
|---|---|---|---|---|---|---|---|
| CFW (♂) | 1 | 1 | 1 | 3 | i.p. | 14 D | Caecum—angiosarcoma. |
| (12) | 1 | 1 | 1 | 3 | i.p. | 14 K | Adrenal cortical tumour. |
| C3H (♂) | 0 | 6 mth | | | | | |
| (24) | 1 | 3 | — | 3 | i.p. | 9·5 K | Liver—hepatoma. |
| | 1 | 2 | — | 3 | i.p. | 13 D | Caecum—adenocarcinoma; Liver and spleen—haemangiomatous changes. |
| | 1 | 2 | — | 3 | i.p. | 13 K | Ileum—adenoma. |
| | 1 | 2 | — | 3 | i.p. | 16 K | Jejunum—adenocarcinoma. |
| | 1 | 2 | — | 3 | i.p. | 16 K | Liver—hepatoma; Lung—adenoma. |
| | 1 | 2 | — | 3 | i.p. | 16 K | Liver—hepatoma; Lung—adenoma. |
| | 1 | 2 | — | 3 | i.p. | 16 K | Liver—hepatoma; Lung—adenoma. |
| | 2·5 | — | — | 2·5 | i.g. | 11 D | Keratinising squamous cell. |
| | 2·5 | — | — | 2·5 | i.g. | 11 K | Somach—squamous cell carcinoma. |
| | 2·5 | — | — | 2·5 | i.g. | 14 D | Stomach—squamous cell carcinoma. |
| | 2·5 | — | — | 2·5 | i.g. | 20 K | Small bowel—adenoma; Liver—adenoma. |
| | 2·5 | — | — | 2·5 | i.g. | 21 K | Liver—polycystic appearance; Lung—adenoma; Stomach—squamous papilloma; Lymph node-reticulum cell tumour. |
| | 1·2 | — | — | 1·2 | s.c. | 17 D | Lung adenoma. |
| | 1·2 | — | — | 1·2 | s.c. | 20 D | Lung adenoma. |
| | 1·2 | — | — | 1·2 | s.c. | 20 K | Lung adenoma. |
| | 1·2 | — | — | 1·2 | s.c. | 21 K | Liver—hepatoma; Lung—adenoma. |
| | 1·2 | — | — | 1·2 | s.c. | 21 K | Liver—hepatoma; Lung—adenoma. |

2. Among the 12 C3H mice given MNG by the intraperitoneal route, one showed a caecal adenocarcinoma with multiple angiomatous changes in the liver and spleen. The jejunum was the site of an adenocarcinoma (Fig. 6) in one case and the ileum the site of a sessile adenoma in another. Small hepatomata and pulmonary adenomata were present in 4 mice.

3. Among 6 C3H mice given MNG by stomach tube, 3 developed squamous carcinomata (Fig. 7), one had a papilloma of the stomach, one had an adenoma of the small bowel, one had a lung adenoma and another a hepatomata.

4. Among 6 C3H mice given a single dose of MNG subcutaneously, 5 had lung adenomas and 1 had a hepatoma.

TABLE III.—*Significant Lesions Found in Rats Treated With* N-*ethyl*-N-*nitroso*-N′-*nitroguanidine*

| Sex (total treated 24) | (Dose (mg./rat) at times of treatment: 0 | 11 days | 28 days | 10 mth | Total dose (mg./rat) | Route | Survival (months) | Significant pathology. |
|---|---|---|---|---|---|---|---|---|
| ♀ | 5 | — | — | — | 5 | i.g. | 16·5 | Polyarteritis. |
| ♀ | 2·5 | 2·5 | 5 | 5 | 15 | i.g. | 24 K | Breast—cystic fibro-adenosis. |
| ♀ | 5 | — | — | — | 5 | i.g. | 24 K | Breast—adenocarcinoma. |
| | 0 | 5 mth | 7 mth | 9 mth | | | | |
| ♂ | 5 | 20 | — | 16 | 41 | i.g. | 14 K | Stomach—warty papillomata. |
| ♂ | 5 | 20 | 20 | 16 | 61 | i.g. | 26 K | Stomach—warty papillomata. |
| | 0 | 6 mth | 9 mth | 10 mth | | | | |
| ♂ | 3 | 4·5 | 5 | 5 | 17·5 | i.p. | 11 K | Ileum—polypoid adeno-carcinoma. |
| ♂ | 3 | 4·5 | 5 | 5 | 17·5 | i.p. | 14 K | Ileum—adenomatous polyp. |
| ♂ | 3 | 4·5 | 5 | 5 | 17·5 | i.p. | 24 K | Lung—anaplastic tumour; Liver—hepatoma. |
| ♂ | 3 | 4·5 | 5 | 5 | 17·5 | i.p. | 20 D | Subcutaneous fibroma. |

B. N-*ethyl*-N-*nitroso*-N′-*nitroguanidine* (*ENG*)

*In Table III are summarised the dosage patterns and times of survival of rats which had significant lesions.*

1. No gastro-intestinal tumours, benign or malignant were found in the female rats given ENG by stomach tube. In the mammary tissues of 2 rats, cystic fibradenosis was seen in one rat and in the other, an adenocarcinoma.

In 2 of the 6 male rats given ENG by stomach tube, warty papillomata of the stomach were present.

2. Of the 12 male rats given intraperitoneal injections of ENG, one was found to have a very large adenocarcinoma near the caecum. Histologically, this tumour was found to contain metaplastic bone (Fig. 8 and 9). A small adenomatous polyp was noted in another rat, but no gastric tumours were seen. One rat, the longest survivor in the group, showed an anaplastic lung tumour together with a liver hepatoma. One rat had a large subcutaneous fibroma.

*Mice*

1. No gastro-intestinal tumours were found in the CFW mice. One mouse had a widespread reticulum-cell tumour with evidence of pulmonary adenomatosis.

2. No gastro-intestinal lesions were seen in the C3H mice given ENG. One mouse had a hepato-cellular carcinoma and 6 others showed both hepatomata and pulmonary adenomata.

Although we have made no mention of a control series of rats and mice, it has been our experience over several years in this laboratory that spontaneous gastro-intestinal tumours are extremely rare (compare: Stewart *et al.*, 1961; Rowlatt, 1967).

### DISCUSSION

Both compounds, MNG and ENG, are sparingly soluble in water and inorganic solvents. They have been administered, therefore, as suspensions of crystals, making the estimation of the dose only approximate. Having found previously that the related alkylnitrosourethanes can induce gastro-intestinal tumours with one or a few doses (Schoental, 1961; Schoental and Magee, 1962; Schoental and Bensted, 1968), it appeared of interest to test whether the same applied to the respective alkylnitrosoguanidines. The present experiments, in which the minimal dosage able to induce tumours was explored, show that both MNG and ENG are carcinogenic, and that, as in the case of the alkylnitrosourethanes, the methyl compound is the more effective carcinogen.

The relative frequency of squamous gastric lesions, benign and malignant in our animals given suspensions of MNG by stomach tube compares well with the results of Craddock (1968*a*), who observed squamous carcinomata of the stomach in all the 6 rats given one to four doses of MNG (100 mg./kg. body weight each) as a suspension. However, the tumours described by Sugimura and Fujimura (1967), who administered MNG as dilute solutions in drinking water (33 or 83 mg. per litre) continuously, for periods up to 1 year, appeared to be exclusively of the glandular stomach. The factors responsible for these differences in tumour type are not yet clear. Whilst we have no direct evidence, the gross appearance of the stomach (Fig. 5) suggests multifocal derivation of the squamous tumours which might be compatible with the deposition of MNG particles in these areas.

Apart from this fact, dose differences may play a part. On the assumption that a rat will drink about 10 ml. of water per day, the total ingestion of MNG over a

---

### EXPLANATION OF PLATES

FIG. 1.—Squamous and adenocarcinomatous gastric tumours in a female rat killed 24 months after the first of five intragastric doses of MNG. H. and E. × 3.

FIG. 2.—Mucinous adenocarcinoma of the glandular part of the stomach shown in Fig. 1. (Arrow) H. and E. × 30.

FIG. 3.—Highly invasive squamous cell carcinoma of the stomach associated with a thymic tumour in a female rat, killed 24 months after the first of three intragastric doses of MNG. The liver showed sub-capsular haemangiomata and severe centri-lobular atrophy. × 1·25.

FIG. 4.—Liver and abdominal mass from a female rat killed 20 months after the first of three intragastric doses of MNG. Note the polycystic appearance of the liver. Kidney showed no cystic changes. The abdominal mass proved to be a fibrosarcoma of low grade malignancy.

FIG. 5.—Stomach of a male rat killed 14 months after the first of three intragastric doses of MNG, showing extreme warty papillomatous appearance of the squamous part.

FIG. 6.—Infiltrating adenocarcinoma of the small intestine of a C3H mouse, associated with an intussusception, killed 16 months after the first of two doses of MNG. H. and E. × 27.

FIG. 7.—Keratinising squamous cell carcinoma of the stomach of a male C3H mouse killed 11 months after a single intragastric dose of MNG. H. and E. × 16.

FIG. 8.—A large well-differentiated polypoid carcinoma of the terminal ileum in a rat killed 11 months after the first of four intraperitoneal doses of ENG.

FIG. 9.—High power view of the tip of tumour depicted in Fig. 8. to show the metaplastic bone present in the tumour stroma. H. and E. × 25.

1

2

Schoental and Benstead.

3

Schoental and Bensted.

4

5

Schoental and Bensted.

6

7

Schoental and Bensted.

8

9

Schoental and Bensted.

period of 12 months in experiments of Sugimura and Fugimura (1967) would be approximately 120 mg. or 300 mg. per rat. This is over 3–15 times the total amount of MNG administered to our rats.

The occurrence of cystic biliary lesions in the liver (Fig. 4) after intragastric administration of suspensions of MNG was described also by Craddock (1968).

MNG has been reported to induce sarcomata in rats, at the site of repeated subcutaneous injections. The total dosage corresponded to 10 or 25 mg. per rat given in 10 weekly doses (Sugimura, Nagao and Okada, 1966) and 225 or 450 mg./kg. body weight given in 5 weekly doses (Druckrey *et al.*, 1966).

There have not yet been reports of testing MNG and ENG in mice. The dosage in the present experiments was minimal; the development of a few gastro-intestinal tumours with MNG indicates that mice are also susceptible to its carcinogenic action. The hepatomas and lung adenomas found in C3H mice cannot, however, be attributed to the action of MNG (or ENG). This strain of mice is known to develop spontaneously a high incidence of hepatomas and some lung adenomas (Heston, Vlahakis and Deringer, 1960; compare also Hoag, 1963).

ENG induced in rats tumours of the intestines with intraperitoneal injections of as little as 17·5 mg./rat; it is of interest that one rat had a hepatoma and another a fibrosarcoma in this series. The latter might have been related to the needle tract.

No gastro-intestinal tumours have been seen in female rats given doses not exceeding 15 mg./rat *in toto*; 9 warty papillomas were found in the stomachs of two rats that had a total dosage of 41 and 61 mg. ENG/rat, respectively.

From these experiments it is obvious that the alkylnitrosoguanidines, MNG and ENG exert similar carcinogenic effects to the alkylnitrosourethanes. The biological similarity of action has its counterpart in their chemical reactivities, especially as regards free thiols. It is of interest that at about pH 6 MNG interacts with thiol groups and this pH has been found to be optimal for its mutagenic action (Adelberg, Mandel and Chen, 1965; Süssmuth and Lingens, 1968, 1969).

The similarity of action extends also to the ability of both MNU and MNG to alkylate nucleic acids mainly at the 7-position of guanine (Schoental, 1967 Craddock, 1968*b*). The extent of alkylation of DNA *in vitro* has been reported to increase in the presence of cysteine with both, MNU (Schoental, 1967) and MNG (Lawley, 1968; McCalla, 1968; Süssmuth and Lingens, 1968). However, the significance of the alkylation of nucleic acids for the mechanism of their carcinogenic action is not clear; no correlation could be found between the extent of alkylation by MNU of DNA or RNA in various rat organs *in vivo* and the localisation of tumours induced by this compound (Schoental, 1969).

In view of the strong carcinogenic action of MNG, its use in childhood leukaemias, or as an antimalarial agent (Siu, 1968) is not advisable.

### SUMMARY

Alkylnitrosoguanidines, the methyl and the ethyl homologues induce in rats mainly tumours of the stomach, when given intragastrically, and tumours of the intestines when injected intraperitoneally. Mice are also susceptible to these compounds, which, like the respective alkylnitrosourethanes, yield an alkylating entity on interacting with sulphydryls, without the need of enzymic activation.

We thank Mr. R. F. Legg for the microphotographs and Mrs Nina Marks for valuable technical assistance.

### REFERENCES

ADELBERG, E. A., MANDEL, M. AND CHEN, P. C. C.—(1965) *Biochem. biophys. Res. Commun.*, **18**, 788.
CRADDOCK, V. M.—(1968*a*) *Experientia*, **24**, 1148.—(1968*b*) *Biochem. J.*, **106**, 921.
DRUCKREY, H., PREUSSMANN, R., IVANKOVIC, S., SO, B. T., SCHMIDT, C. H. AND BÜCHELER, J.—(1966) *Z. Krebsforsch.*, **68**, 87.
EISENSTARK, A., EISENSTARK, R. AND SICKLE, R. V.—(1965) *Mutation Res.*, **2**, 1.
GICHNER, T., MICHAELIS, A. AND RIEGER, R.—(1963) *Biochem. biophys. Res. Commun.*, **11**, 120.
GICHNER, T. AND VELEMINSKÝ, J.—(1967) *Mutation Res.*, **4**, 207.
GILHAM, N. W.—(1965) *Genetics, Princeton*, **52**, 529.
GOLDIN, A., VENDITTI, J. M. AND KLINE, I.—(1959) *Cancer Res.*, **19**, 429.
GREENE, M. O. AND GREENBERG, J.—(1960) *Cancer Res.*, **20**, 1166.
HESTON, W. E., VLAHAKIS, G. AND DERINGER, M. K.—(1960) *J. natn. Cancer Inst.*, **24**, 425.
HOAG, W. G.—(1963) *Ann. N.Y. Acad. Sci.*, **108**, 805.
KAUL, B. L.—(1969) *Mutation Res.*, **7**, 43.
LAWLEY, P. D.—(1968) *Nature, Lond.*, **218**, 581.
LINGENS, F. AND OLTMANNS, O.—(1966) *Z. Naturforsch.*, **21b**, 660.
MCCALLA, D. P.—(1965) *Science, N.Y.*, **148**, 497.
MCCALLA, D. P.—(1968) *Biochim. biophys. Acta*, **155**, 114.
MAGEE, P. N. AND BARNES, J. M.—(1967) *Adv. Cancer Res.*, **10**, 163.
MANDELL, J. D. AND GREENBERG, J.—(1960) *Biochem. biophys. Res. Commun.*, **3**, 575.
MÜLLER, A. J. AND GICHNER, T.—(1964) *Nature, Lond.*, **201**, 1149.
ROWLATT, U. F.—(1967) in ' Pathology of Laboratory Rats and Mice ', edited by Cotchin, E. and Rose, F. J. C. Oxford: (Blackwell Scientific Publications) 57.
SCHOENTAL, R.—(1961) *Nature, Lond.*, **192**, 670.—(1963) *Nature, Lond.*, **199**, 190.—(1966) *Nature, Lond.*, **209**, 726.—(1967) *Biochem. J.* **102**, 5C.—(1968) *in Japanese Cancer Association Gann Monograph* **3**, 61 (Maruzen Co., Tokyo).—(1969) *Biochem. J.* in press.
SCHOENTAL, R. AND BENSTED, J. P. M.—(1968) *Br. J. Cancer*, **22**, 316.
SCHOENTAL, R. AND MAGEE, P. N.—(1962) *Br. J. Cancer*, **16**, 92.
SINGER, B. AND FRAENKEL-CONRAT, H.—(1967) *Proc. natn. Acad. Sci. U.S.A.*, **58**, 234.
SIU, P. M. L.—(1968) *Proc. Soc. exp. Biol. Med.*, **129**, 753.
STEWART, H. L., SNELL, K. C., MORRIS, H. P., WAGNER, B. P. AND RAY, F. E.—(1961) *Natn. Cancer Inst. Monogr.*, **5**, 105.
SUGIMURA, T. AND FUGIMURA, S.—(1967) *Nature, Lond.*, **216**, 943.
SUGIMURA, T., NAGAO, M. AND OKADA, Y.—(1966) *Nature, Lond.*, **210**, 962.
SÜSSMUTH, R. AND LINGENS, F.—(1968) *Naturwissenschaften*, **55**, 85.—(1969) *Z. Naturforsch.*, **24**, 903.


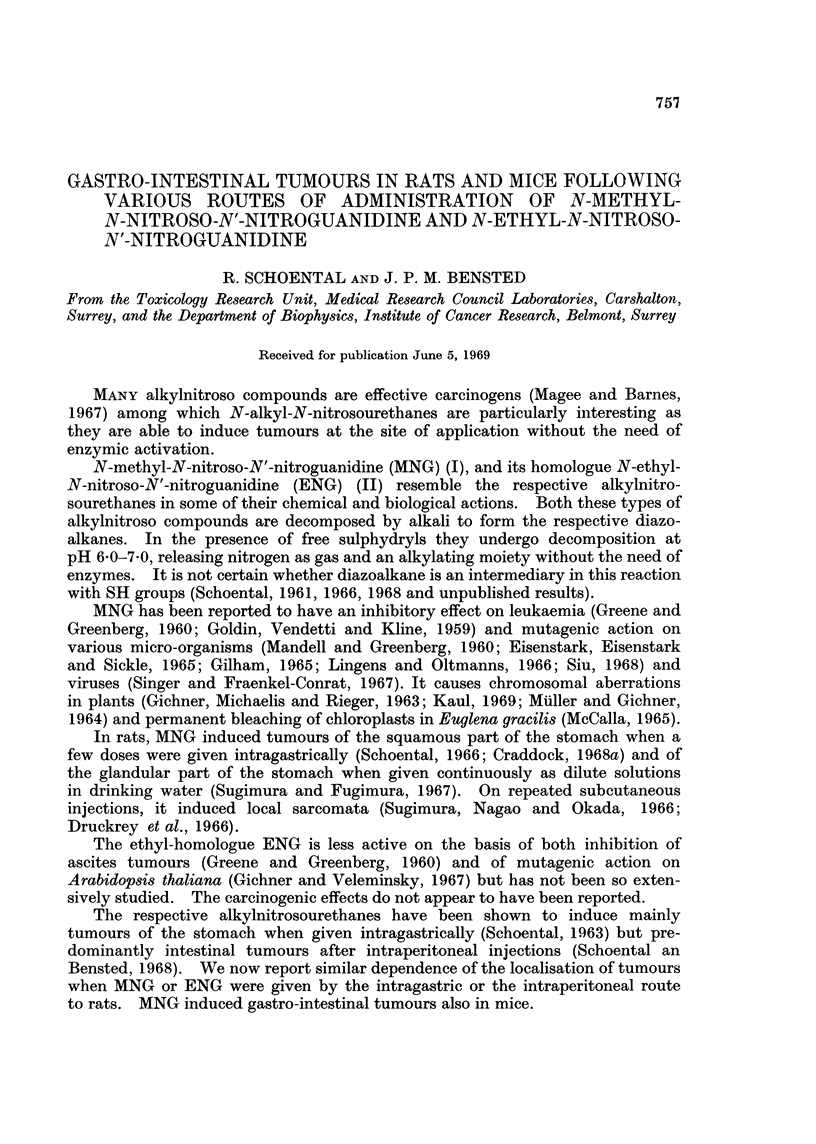

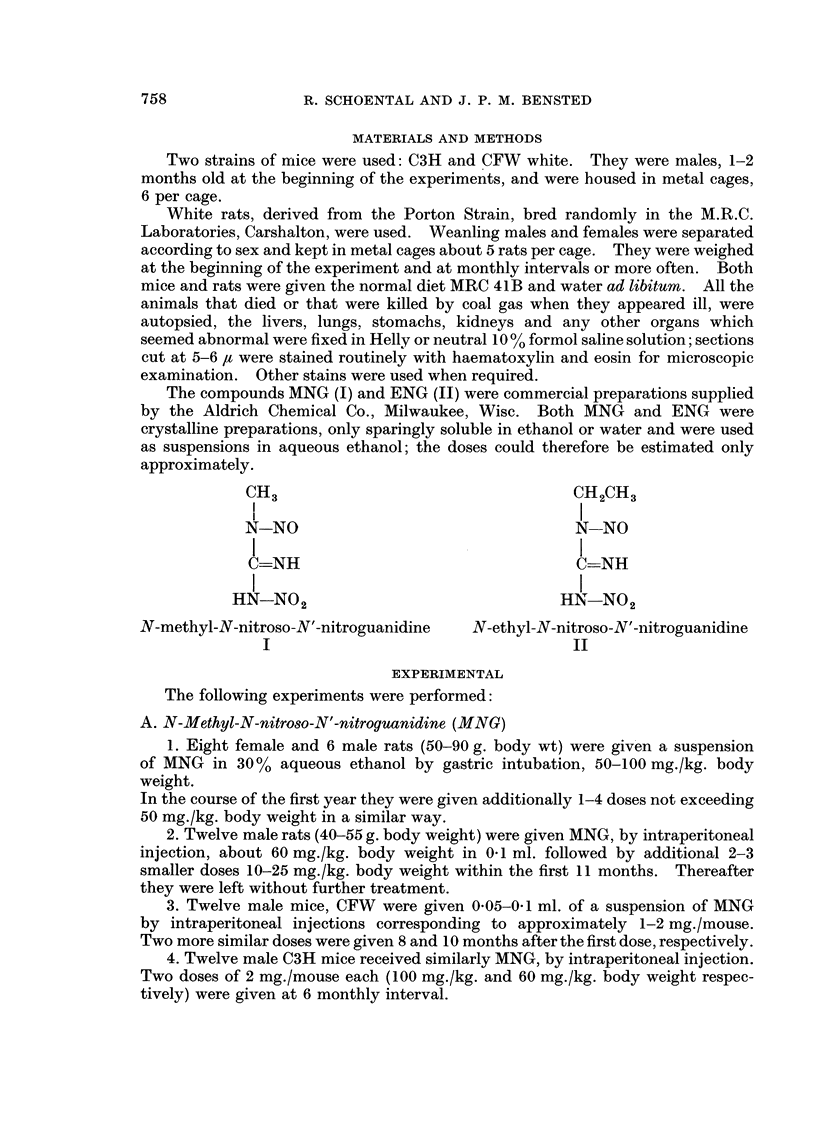

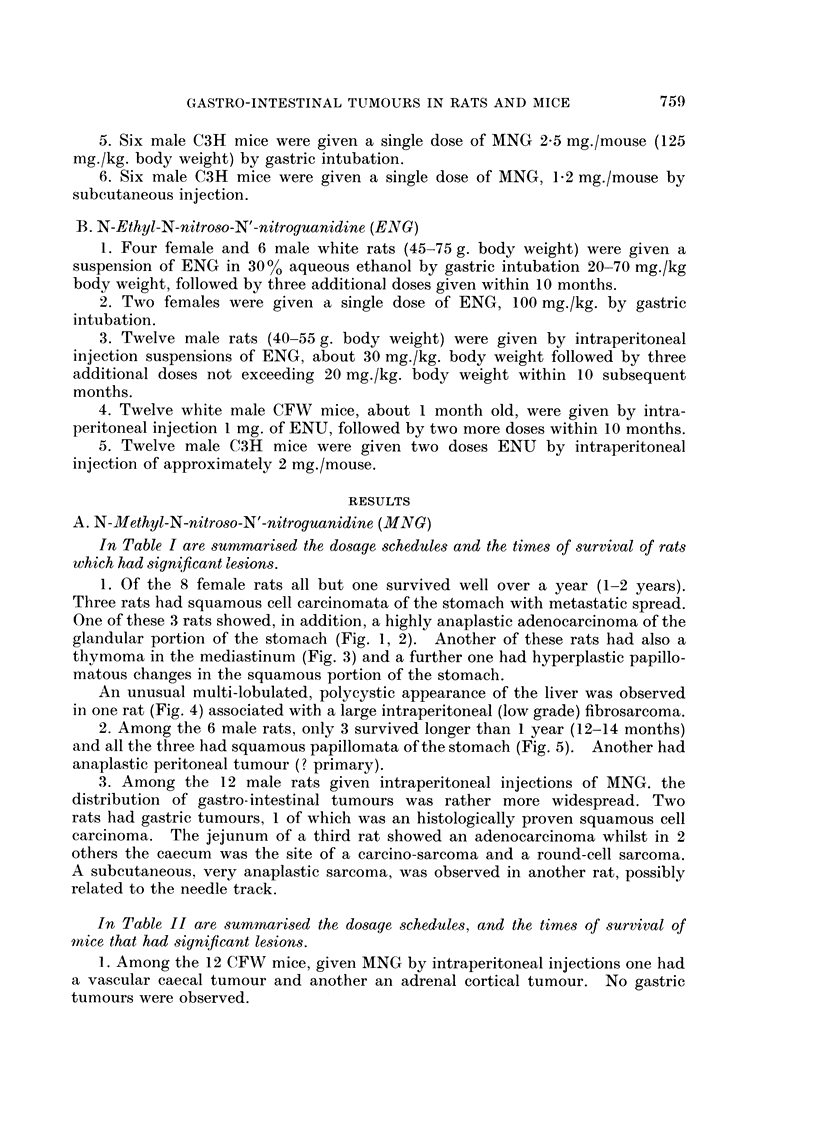

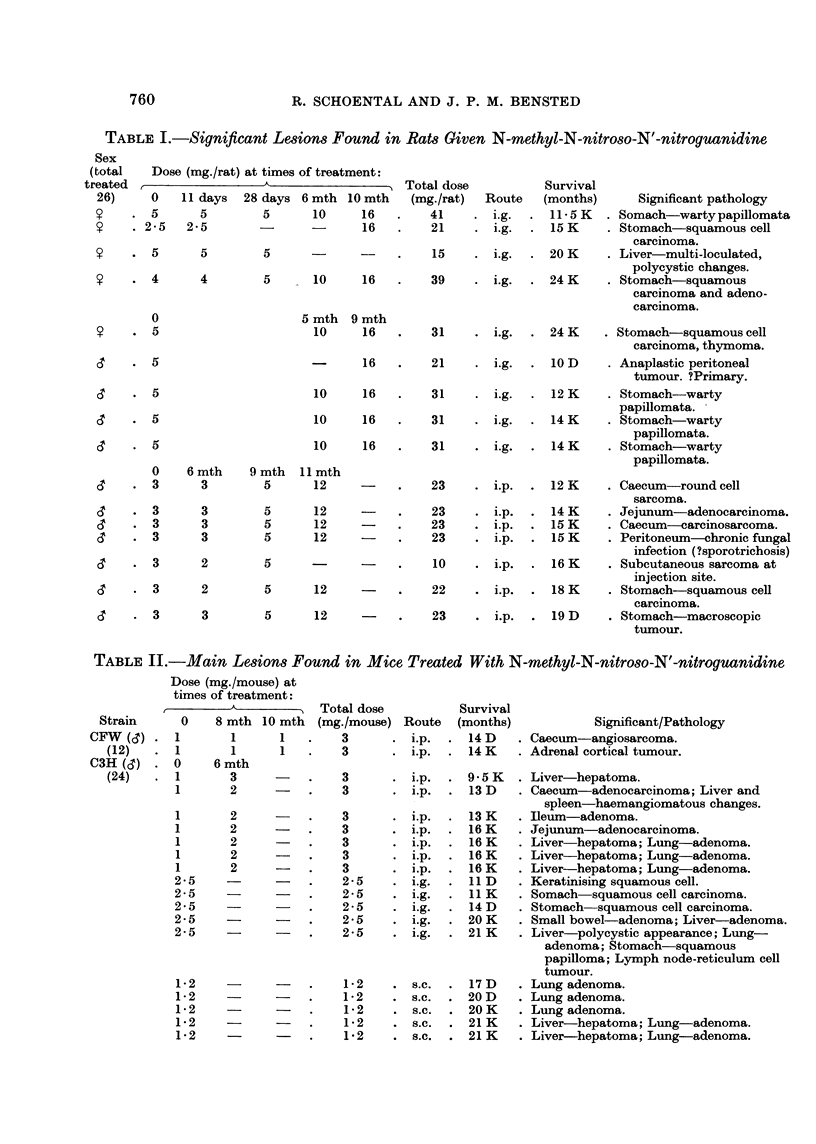

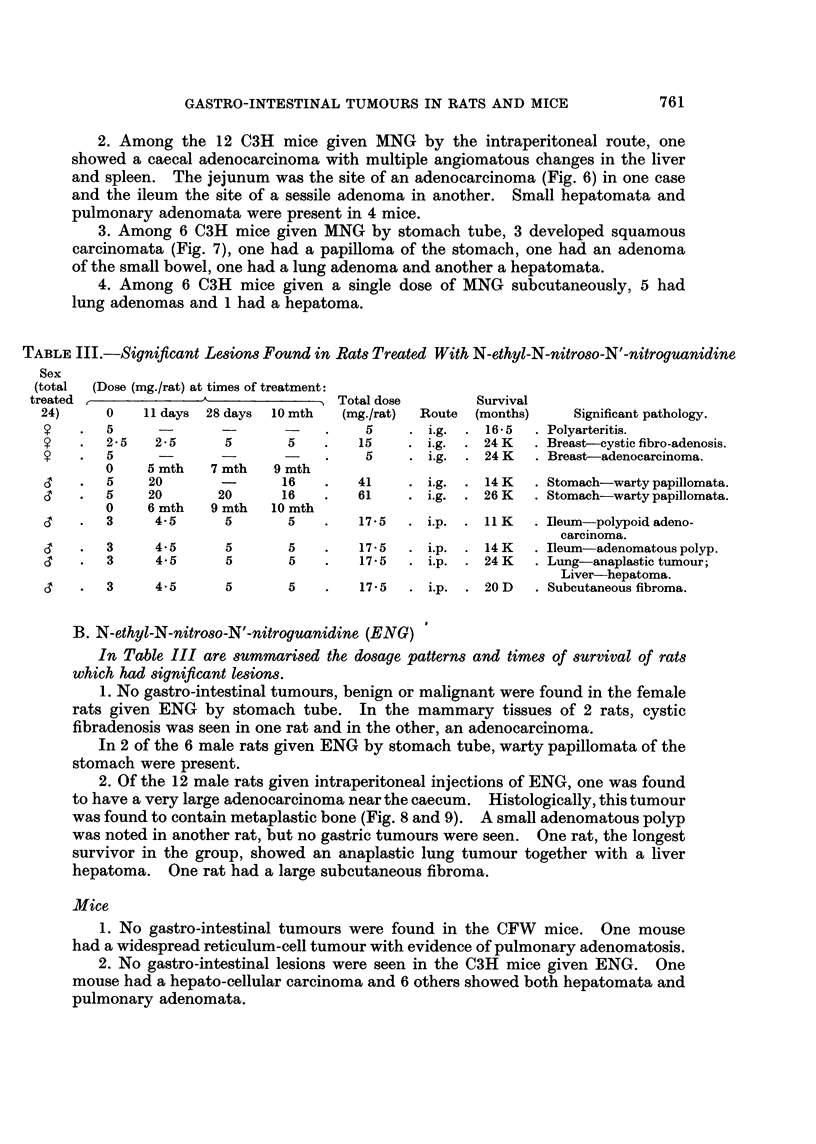

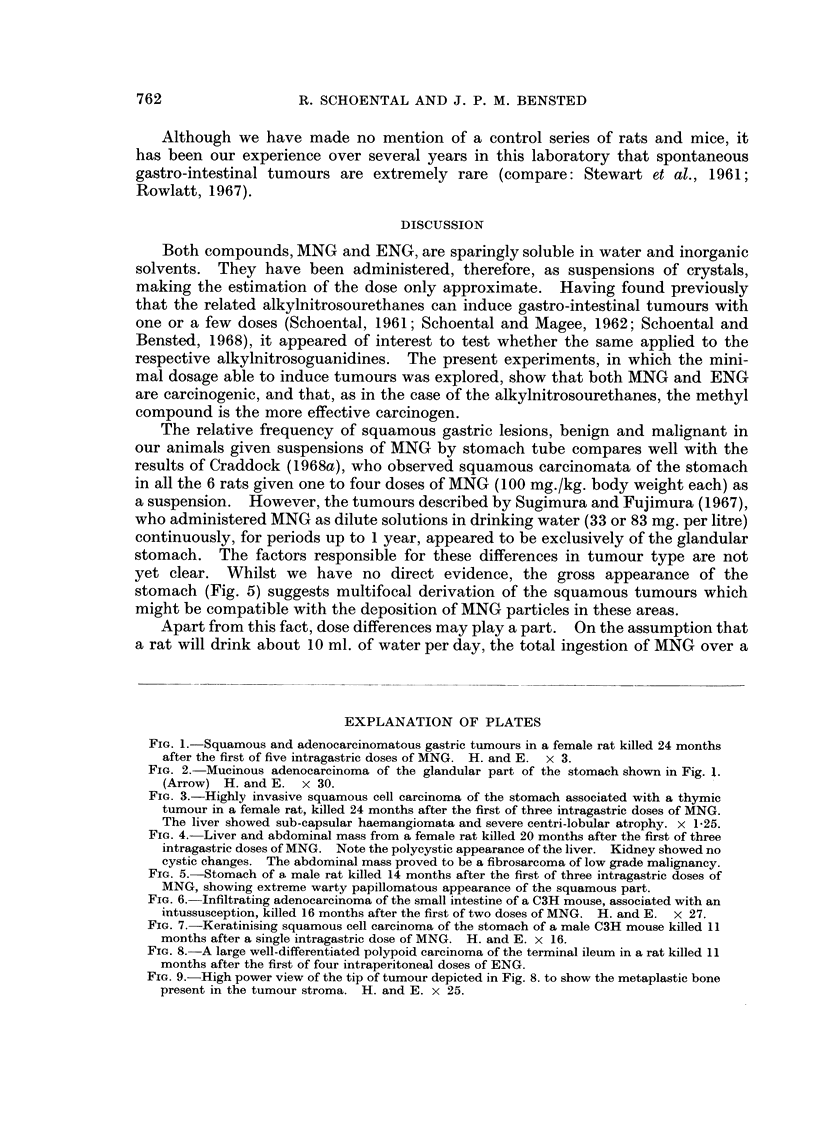

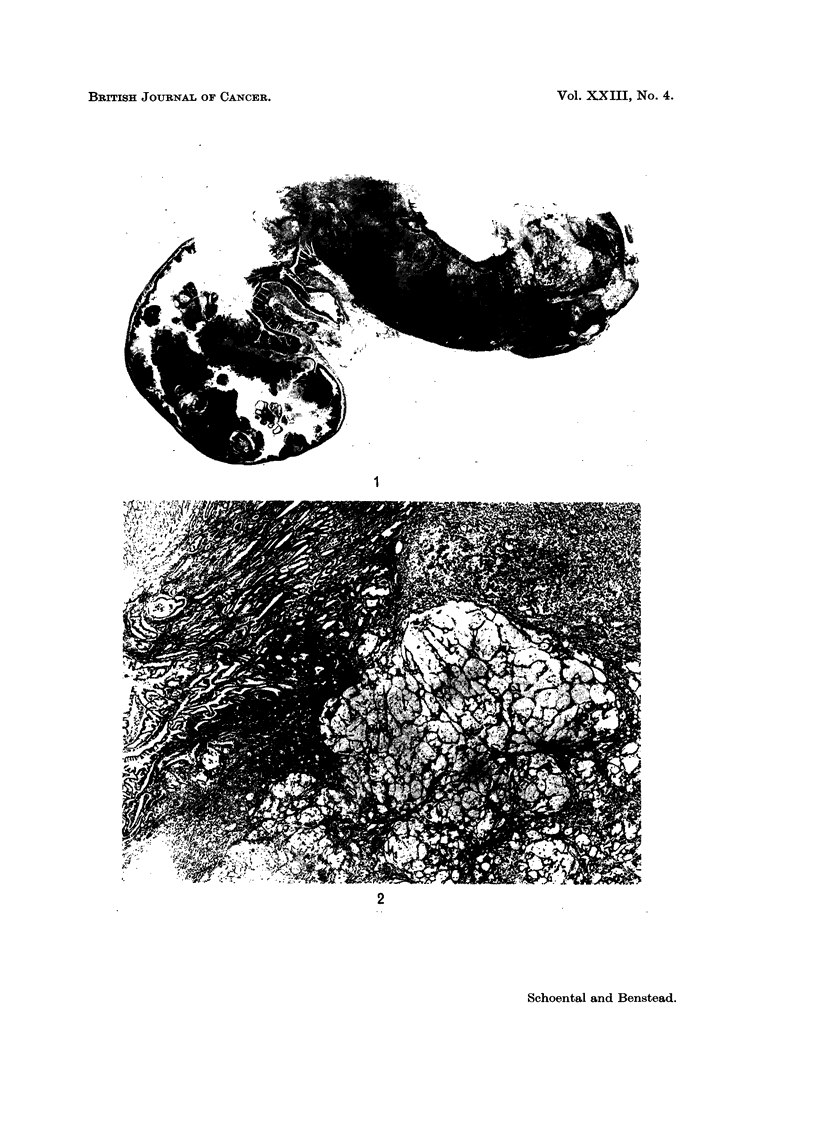

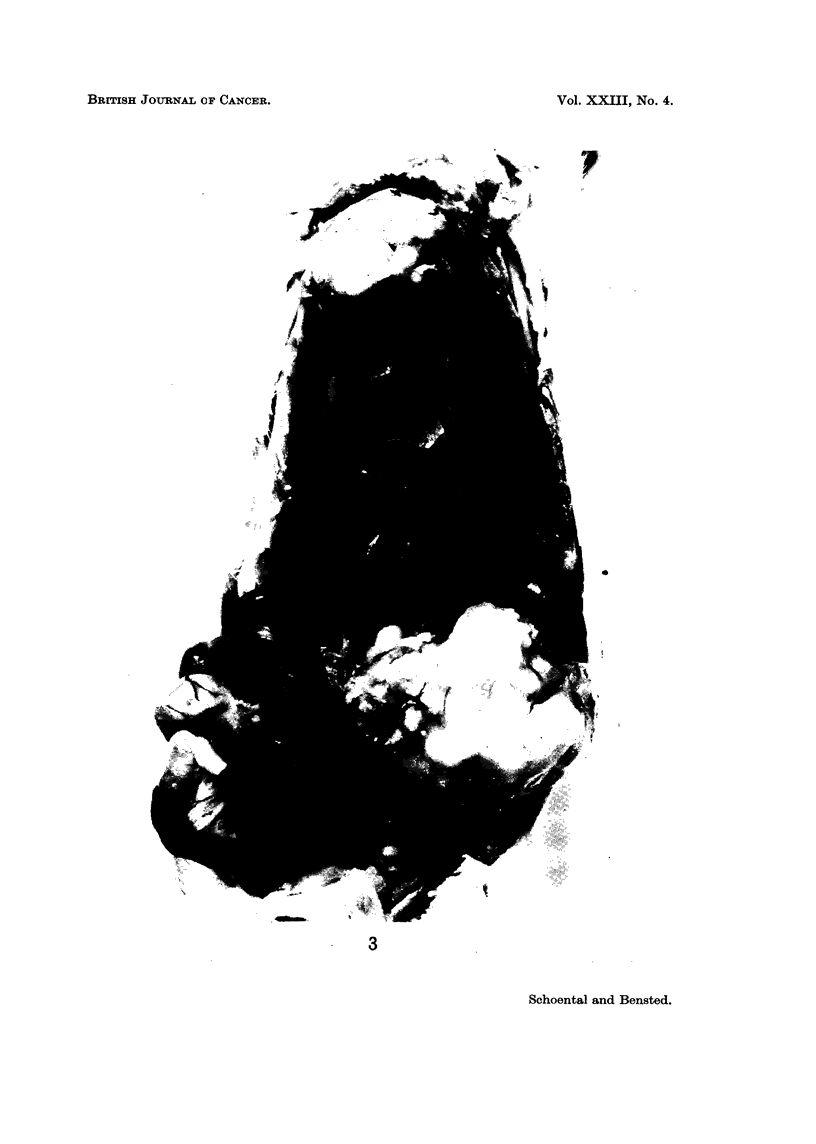

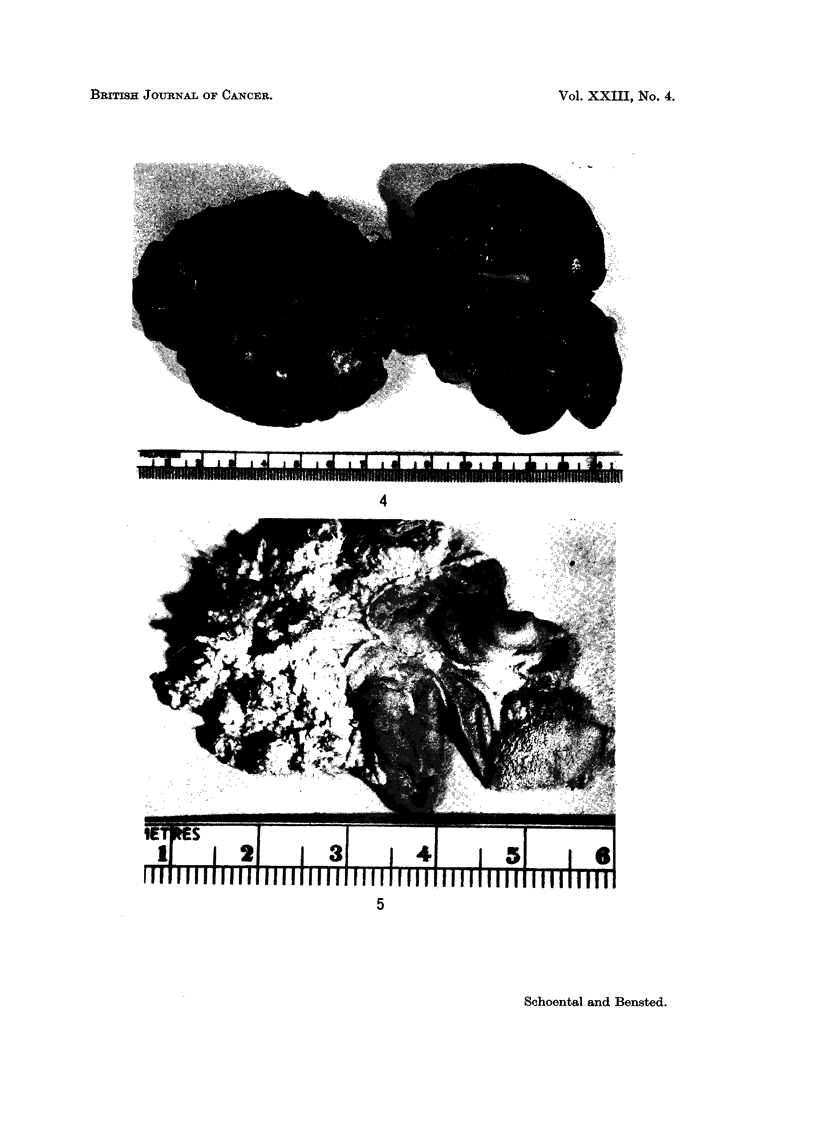

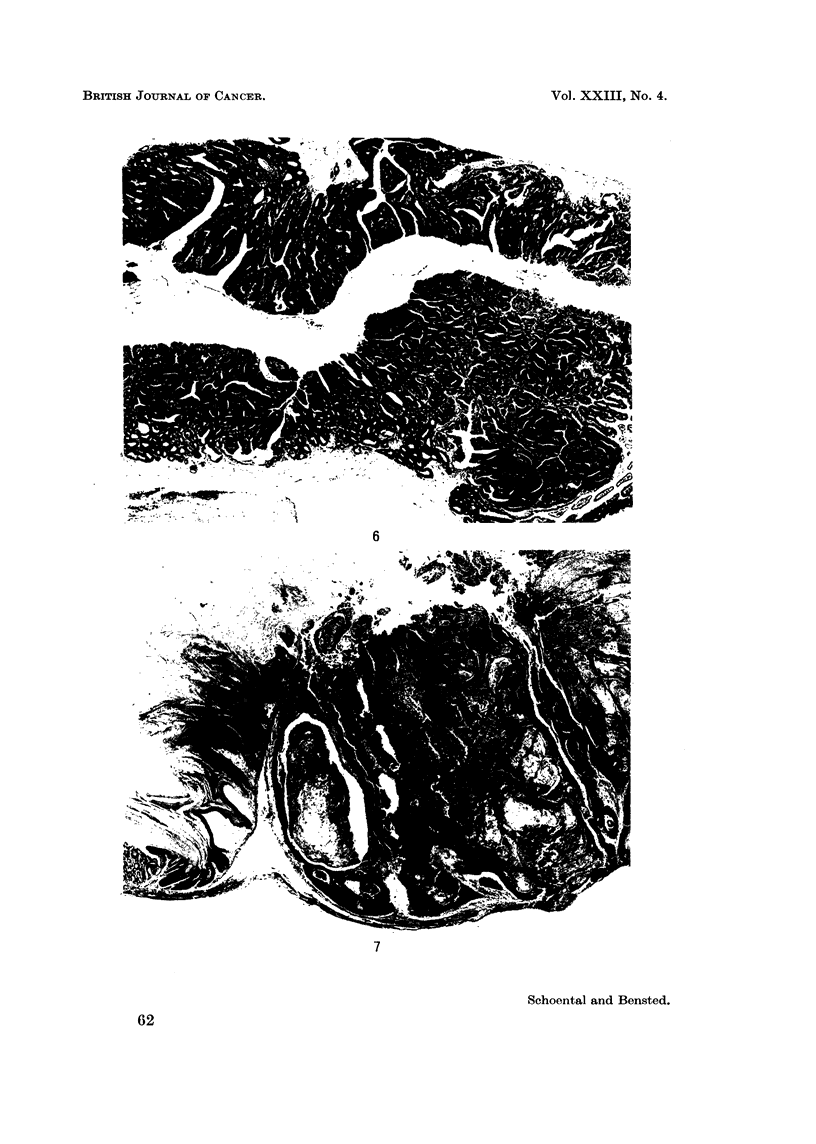

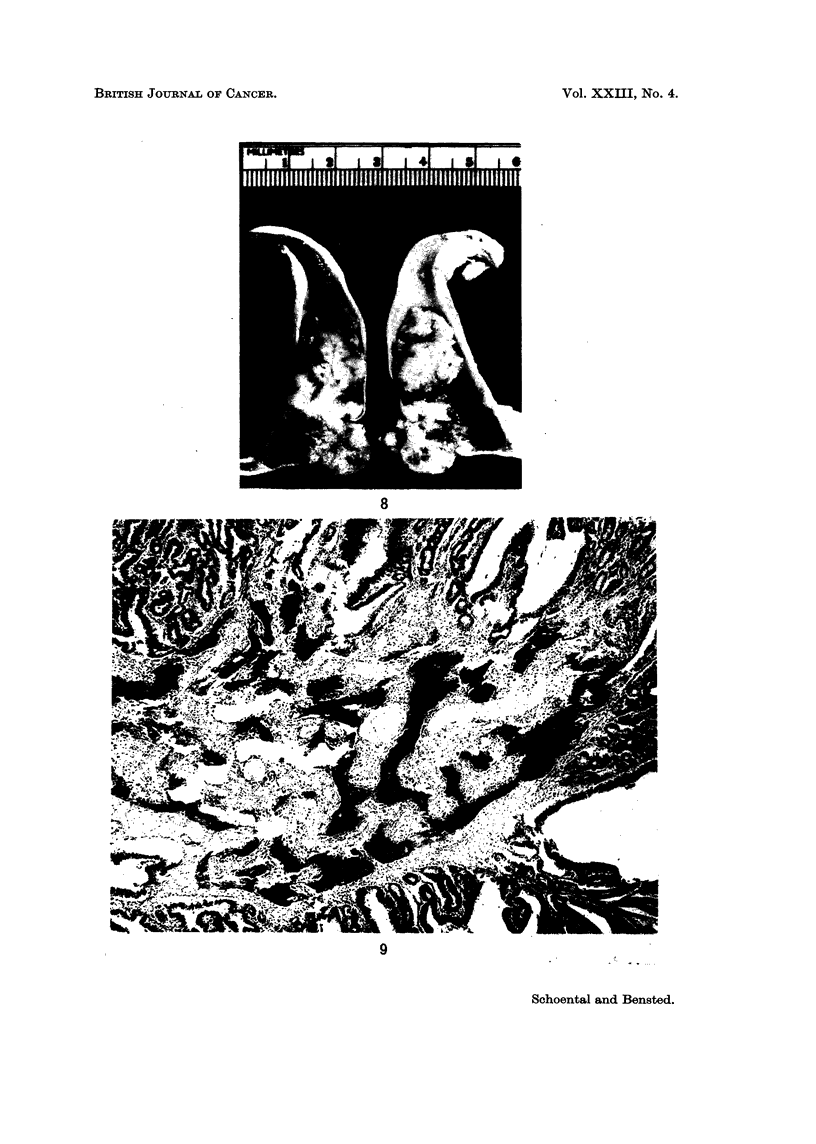

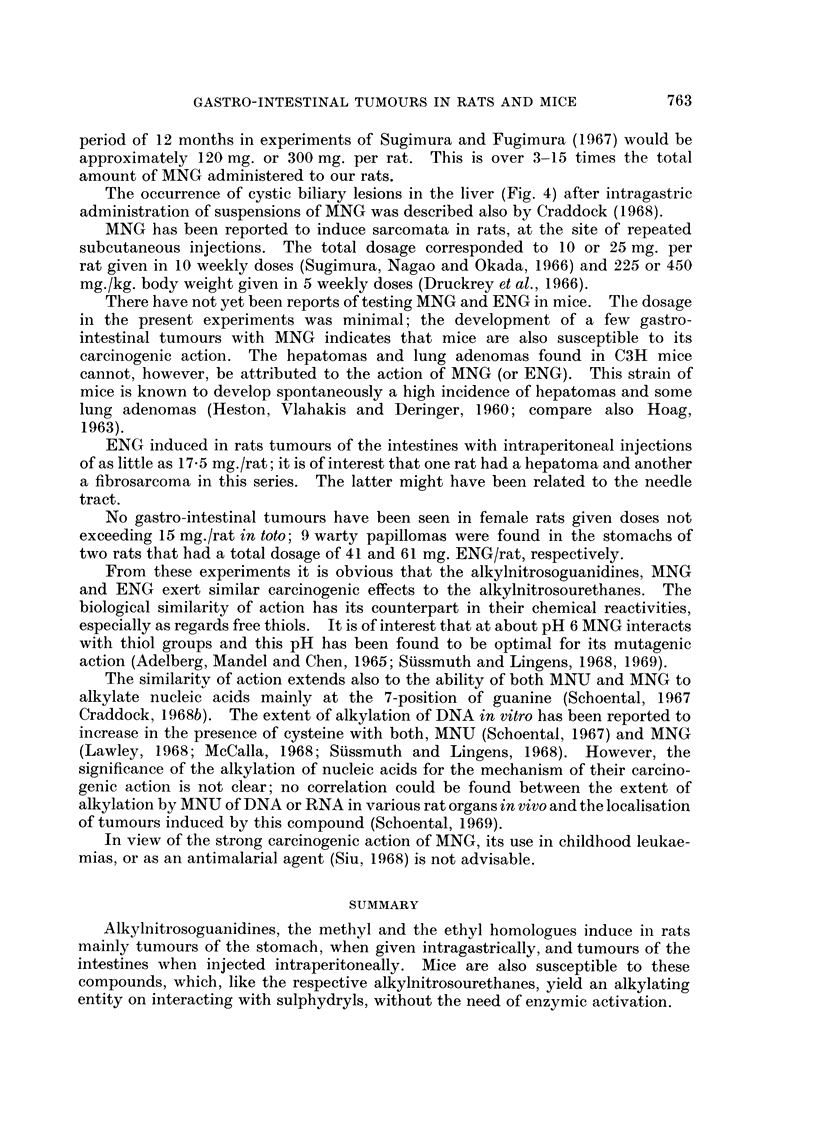

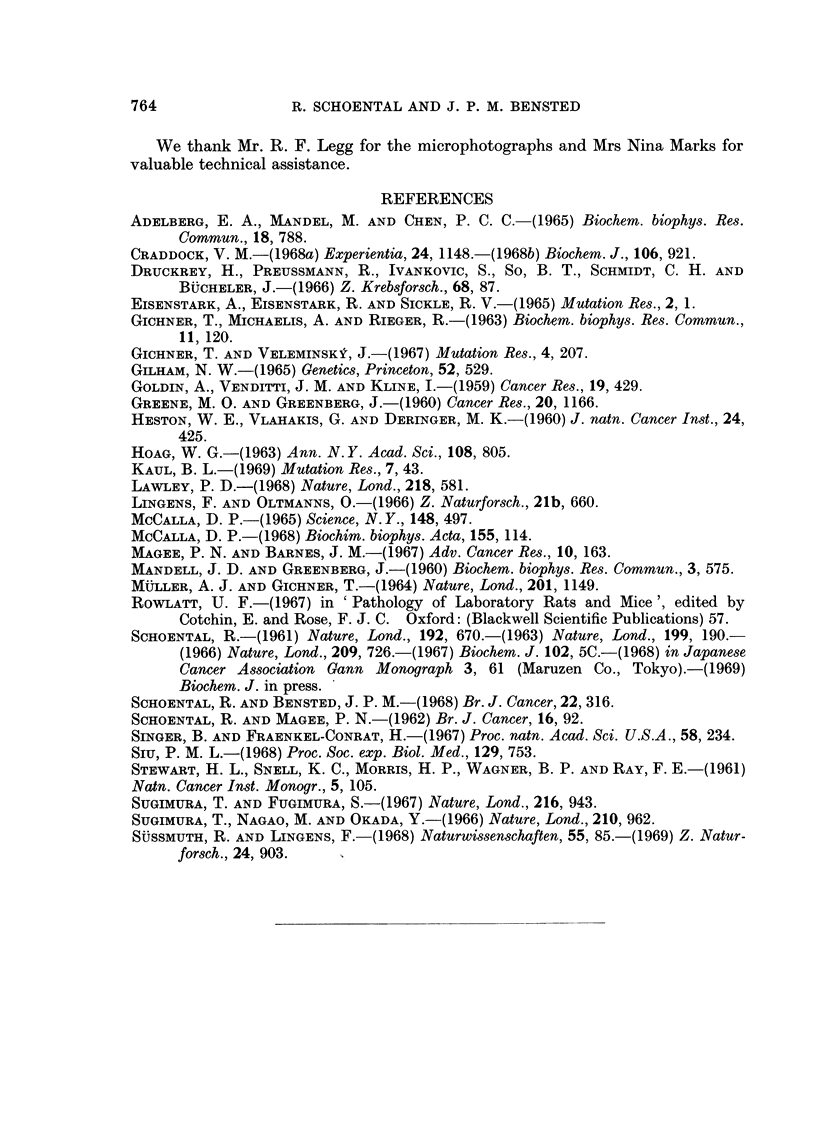


## References

[OCR_00651] Craddock V. M. (1968). The effect of N'-nitro-N-nitrose-N-methylguanidine on the liver after administration to the rat.. Experientia.

[OCR_00665] GREENE M. O., GREENBERG J. (1960). The activity of nitrosoguanidines against ascites tumors in mice.. Cancer Res.

[OCR_00672] HOAG W. G. (1963). SPONTANEOUS CANCER IN MICE.. Ann N Y Acad Sci.

[OCR_00674] Lawley P. D. (1968). Methylation of DNA by N-methyl-N-nitrosourethane and N-methyl-N-nitroso-N'-nitroguanidine.. Nature.

[OCR_00683] MANDELL J. D., GREENBERG J. (1960). A new chemical mutagen for bacteria, 1-methyl-3-nitro-1-nitrosoguanidine.. Biochem Biophys Res Commun.

[OCR_00686] MUELLER A. J., GICHNER T. (1964). MUTAGENIC ACTIVITY OF 1-METHYL-3-NITRO-1-NITROSOGUANIDINE ON ARABIDOPSIS.. Nature.

[OCR_00697] Schoental R., Bensted J. P. (1968). Tumours of the intestines induced in rats by intraperitoneal injections of N-methyl- and N-ethyl-N-nitrosourethanes.. Br J Cancer.

[OCR_00692] Schoental R. (1966). Carcinogenic activity of N-methyl-N-nitroso-N'-nitroguanidine.. Nature.

[OCR_00699] Singer B., Fraenkel-Conrat H. (1967). Chemical modification of viral RNA. VI. The action of N-methyl-N'-nitro-N-nitrosoguanidine.. Proc Natl Acad Sci U S A.

[OCR_00700] Siu P. M. (1968). Antimalarial activity of 1-methyl-3-nitro-1-nitrosoguanidine.. Proc Soc Exp Biol Med.

[OCR_00705] Sugimura T., Nagao M., Okada Y. (1966). Carcinogenic action of N-methyl-N'-nitro-N-nitrosoguanidine.. Nature.

[OCR_00709] Süssmuth R., Lingens F. (1969). Zum Wirkungsmechanismus von 1-Nitroso-3-nitro-1-methylguanidin (NNMG) bei der Mutationsauslösung. IV. Beständigkeit des NNMG, Beziehungen zwischen Mutationsrate und Aufnahme des Mutagens durch die Zelle und Förderung der Methylierung durch Sulfhydrylgruppen in Abhängigkeit vom pH-Wert.. Z Naturforsch B.

